# Near Misses as Signals of System Vulnerability in Thoracic Surgery: A Narrative Review on Quality Improvement and Patient Safety

**DOI:** 10.3390/healthcare14040423

**Published:** 2026-02-08

**Authors:** Dimitrios E. Magouliotis, Vasiliki Androutsopoulou, Prokopis-Andreas Zotos, Andrew Xanthopoulos, Ugo Cioffi, Noah Sicouri, Piergiorgio Solli, Marco Scarci

**Affiliations:** 1 Department of Cardiac Surgery Research, Lankenau Institute for Medical Research, Wynnewood, PA 19096, USA; 2 Department of Cardiology, University of Thessaly, Biopolis, 41110 Larissa, Greece; andrewvxanth@gmail.com; 3 Department of Cardiothoracic Surgery, University of Thessaly, Biopolis, 41110 Larissa, Greece; androutsopoulouvasiliki@uth.gr (V.A.); zotospro@hotmail.com (P.-A.Z.); 4 Department of Surgery, University of Milan, 20157 Milan, Italy; ugo.cioffi@guest.unimi.it; 5 Department of Neuroscience Pittsburgh Campus, University of Pittsburgh, Pittsburgh, PA 15260, USA; nps67@pitt.edu; 6 Department of Thoracic Surgery, Fondazione IRCCS Istituto Nazionale dei Tumori Milano, Via Giacomo, Venezian 1, 20133 Milan, Italy; piergiorgio.solli@gmail.com; 7 Department of Cardiothoracic Surgery, Hammersmith Hospital, Imperial College Healthcare, National Health Service (NHS) Trust, London W2 1NY, UK; marco.scarci@nhs.net

**Keywords:** thoracic surgery, near misses, quality improvement, failure to rescue, patient safety

## Abstract

Near misses—clinical events that could have resulted in patient harm but did not—are increasingly recognized as critical yet underutilized sources of insight in surgical quality improvement. In thoracic surgery, where procedures are physiologically demanding and care pathways are highly interdependent, near misses frequently precede major complications and expose latent system vulnerabilities rather than isolated technical errors. A structured narrative review methodology was employed, including a targeted literature search of major biomedical databases and thematic synthesis of relevant studies. This narrative review synthesizes evidence from patient safety science, surgical quality literature, and thoracic surgery—specific outcomes research to examine how near misses can be systematically leveraged to improve care. We discuss the transition from individual-centered explanations of adverse events to system-based models that emphasize human factors, communication, escalation pathways, and organizational culture. Particular attention is given to contemporary quality frameworks such as failure to rescue and textbook outcome, which highlight the importance of early recognition, coordinated response, and recovery from complications rather than complication avoidance alone. We further explore the central role of psychological safety and leadership behaviors in enabling meaningful learning from near misses. By reframing near misses as actionable data rather than anecdotal “close calls,” quality improvement emerges as a core professional responsibility in thoracic surgery. We conclude that excellence in thoracic surgery should be defined not by the absence of complications, but by the capacity of surgical systems to learn, adapt, and prevent future harm.

## 1. Introduction

Thoracic surgery represents one of the most complex domains of modern surgical practice, characterized by high physiological risk, technical variability, and reliance on tightly coupled multidisciplinary care pathways. Despite substantial advances in surgical technique, perioperative management, and risk stratification, adverse events remain an inherent feature of thoracic surgical care. Of note, many adverse outcomes are preceded by near misses—clinical events in which patient harm is narrowly avoided—yet these events remain inconsistently recognized, reported, and systematically analyzed [1,2,3]. The landmark report To Err Is Human catalyzed a paradigm shift by reframing medical error as a systems problem rather than a consequence of individual failure, emphasizing that high-quality care cannot rely solely on professional expertise but must be supported by organizational structures, processes, and cultures designed to anticipate failure and mitigate its consequences [1,2]. Within this framework, near misses have emerged as particularly valuable signals of system vulnerability, offering insight into how care delivery fails safely before irreversible harm occurs [3,4,5].

In thoracic surgery, near misses commonly arise at the interface between technical care and system performance, including delayed recognition of postoperative respiratory deterioration, breakdowns in escalation pathways, and communication failures during transitions of care. Although such events are often attributed retrospectively to individual oversight or clinical judgment, patient safety science consistently demonstrates that they reflect latent system conditions—such as workflow design, human factors, and organizational culture—rather than isolated errors [5,6,7]. Traditional mechanisms for learning from adverse events, particularly morbidity and mortality conferences, have played an important educational role but remain limited in their ability to generate durable system-level improvement. These forums are frequently retrospective and case-centered, with implicit emphasis on individual decision-making and variable attention to the contextual factors that shape clinical behavior, allowing similar near misses to recur across patients and providers without systematic learning [8,9].

Quality improvement (QI) offers a complementary framework that emphasizes systems thinking, continuous learning, and proactive redesign. Rather than asking “what went wrong,” QI focuses on why the system allowed an event to occur and how similar events might be prevented in the future [3,4]. This approach does not seek to eliminate human error—an unrealistic goal in complex clinical environments—but to design systems that are resilient to it [6,7,8,9,10,11,12,13,14,15]. Recent advances in surgical quality measurement further underscore the relevance of near misses. Frameworks such as failure to rescue and textbook outcome, a composite metric reflecting complication-free recovery, absence of mortality, and timely discharge, shift attention away from complication rates alone toward the system’s ability to recognize deterioration, coordinate timely responses, and support recovery following adverse events [16,17,18,19,20,21,22,23,24,25,26]. Within these paradigms, near misses occupy a central position, representing moments when rescue was possible and successful, but not guaranteed. Accordingly, the aim of this narrative review is to synthesize evidence from patient safety science, surgical quality literature, and thoracic surgery-specific outcomes to examine how near misses can be leveraged as actionable data to drive meaningful quality improvement and redefine excellence in thoracic surgery.

## 2. Methods

This article was designed as a narrative (non-systematic) review to integrate evidence and concepts from patient safety science, organizational behavior, and thoracic surgery–specific quality and outcomes literature. A narrative approach was selected because near-miss learning spans heterogeneous study designs and disciplinary frameworks (e.g., safety science, human factors, and surgical outcomes research), making formal systematic synthesis or meta-analysis methodologically inappropriate for the goals of conceptual integration and practice-oriented interpretation.

### 2.1. Search Strategy

A structured literature search was performed in PubMed/MEDLINE, Scopus, and Web of Science from database inception through December 2025. Searches were performed using database-adapted Boolean strategies combining near-miss terminology with patient safety/quality improvement constructs and thoracic surgery keywords. The core search logic included:

(“near miss*” OR “close call*” OR “safety event*” OR “incident reporting” OR “postoperative deterioration”) AND (“patient safety” OR “quality improvement” OR “human factors” OR “resilience” OR “failure to rescue” OR “textbook outcome” OR “escalation”) AND (“thoracic surgery” OR “lung resection” OR “lobectomy” OR “pneumonectomy” OR “cardiothoracic surgery”).

Reference lists of key reports and highly relevant thoracic surgery outcomes studies were manually screened to identify additional eligible sources.

### 2.2. Eligibility Criteria and Study Selection

We included peer-reviewed articles and landmark reports that addressed near misses, system vulnerability, postoperative deterioration/escalation pathways, failure-to-rescue, textbook outcome, resilience, safety culture, or quality improvement, with emphasis on relevance to thoracic surgery and perioperative surgical systems. Articles were excluded if they (i) focused exclusively on non-surgical settings with no transferable system-learning implications, (ii) lacked relevance to near-miss learning or system-level mechanisms, or (iii) were not available in English. Study selection was performed by screening titles/abstracts for relevance followed by full-text review of potentially eligible records.

### 2.3. Search Yield and Synthesis

The search identified approximately 620 records. After removal of duplicates and initial screening, 94 full-text articles were assessed for eligibility and 41 sources were ultimately included for thematic synthesis, spanning patient safety frameworks, quality improvement theory, and thoracic surgery outcomes research. Given the narrative nature of the review, no formal risk-of-bias assessment was undertaken. Evidence was synthesized thematically, focusing on (i) recognition and escalation mechanisms, (ii) system contributors to rescue and recovery, (iii) operationalization through contemporary quality frameworks (failure to rescue, textbook outcome), and (iv) the roles of culture, leadership, and psychological safety in translating near-miss learning into durable improvement.

## 3. Why near Misses Matter: From Individual Error to System Vulnerability

Near misses in thoracic surgery can be defined as clinical events in which a potentially harmful process, such as delayed recognition of deterioration, failure of escalation, or breakdown in coordination, was initiated but intercepted before resulting in permanent patient injury. Near misses are clinically meaningful precisely because they occur at the boundary between safety and harm. In thoracic surgery, these events often present as unexpected yet recoverable deviations from the anticipated postoperative course—such as transient respiratory deterioration, delayed escalation of care, unanticipated air leaks, or narrowly avoided failures to rescue. Although these events may resolve without lasting sequelae, patient safety research has consistently demonstrated that serious adverse outcomes are rarely abrupt; instead, they are typically preceded by smaller, less conspicuous failures that accumulate over time, including missed cues, delayed decisions, and communication lapses [1,5]. Near misses, therefore, represent early manifestations of system stress, offering insight into how complex clinical environments behave under pressure and where margins of safety begin to erode. In the context of thoracic surgery, system vulnerability is particularly consequential because postoperative deterioration often evolves rapidly and non-linearly. Studies of lung resection outcomes demonstrate that institutional variation in mortality is driven more by differences in rescue capacity than by complication rates alone, highlighting the central role of timely recognition, escalation, and coordinated response [16,26]. Near misses frequently occur during this vulnerable window, such as delayed escalation of hypoxia or evolving respiratory failure, and therefore offer clinically meaningful insight into how thoracic surgical systems perform under stress.

Thoracic surgery is particularly susceptible to these dynamics because of the physiological vulnerability of patients, the non-linear nature of postoperative recovery, and the fragmentation of care across multiple settings and teams. The success of thoracic surgical care depends not only on operative technique but also on timely recognition of deterioration, effective communication, and reliable escalation across the operating room, recovery unit, ward, and intensive care unit. When near misses occur, they frequently expose weaknesses at these interfaces rather than deficiencies in individual technical performance [6,7]. However, surgical culture has traditionally minimized such events, framing them as “close calls” attributable to experience or intuition rather than as signals warranting systematic analysis. Safety science cautions that this normalization of deviance—repeated exposure to risk without immediate harm—can gradually reduce vigilance and increase the likelihood that future events will result in irreversible injury [7].

From a quality improvement perspective, near misses matter because they are far more frequent than catastrophic outcomes and therefore provide a richer substrate for learning. Mortality and major morbidity, while essential outcome measures, are relatively infrequent in contemporary thoracic surgery and offer limited insight into everyday system performance. Near misses, by contrast, occur regularly and capture real-time interactions between clinicians, workflows, and organizational structures [8,9]. Of note, they also carry a significant cognitive and emotional burden for surgical teams. Without structured reflection, these experiences may contribute to defensive practice or disengagement from improvement efforts. Conversely, in environments characterized by psychological safety, near misses can strengthen team learning and collective vigilance, transforming uncomfortable experiences into drivers of improvement [10,11,12,13].

Recognizing near misses as meaningful clinical signals requires a deliberate shift in perspective—from asking whether harm occurred to asking why harm was avoided. This reframing aligns with contemporary safety theory, which emphasizes learning from successful recovery and everyday performance rather than from failure alone [23,24]. In thoracic surgery, where resilience often determines outcomes, understanding how teams adapt under uncertainty is as important as cataloging adverse events. Accordingly, near misses should be viewed not as peripheral anecdotes but as central data points that illuminate system vulnerability, guide targeted intervention, and ultimately improve the reliability and safety of thoracic surgical care. A conceptual framework contrasting traditional and systems-based interpretations of near misses and their implications for quality improvement in thoracic surgery is summarized in Table 1.

For much of surgical history, adverse events have been interpreted predominantly through the lens of individual performance. Complications and near misses were commonly attributed to lapses in judgment, technical shortcomings, or insufficient experience, and learning focused on how individual clinicians might act differently in future cases. While this approach has enduring educational value, patient safety research has demonstrated that it provides an incomplete and often misleading explanation for why harm occurs in complex clinical environments [1,3]. Errors in healthcare are now understood to arise from interactions between individuals, tasks, technologies, and organizational context, rather than from isolated acts of human failure [5,6]. Within this framework, human error is not the root cause of adverse events, but a symptom of deeper system vulnerabilities.

In thoracic surgery, near misses frequently emerge from chains of small, individually reasonable actions that align in unintended ways. Delayed escalation of postoperative hypoxia, for example, may reflect ambiguous monitoring thresholds, unclear accountability, competing clinical priorities, or communication failures during handover rather than a single incorrect decision. Retrospective analysis often seeks to identify a discrete “missed opportunity,” yet such simplification obscures the reality that system design shaped both the available choices and the likelihood of error [5,6,7]. Because thoracic surgical care depends on tightly coordinated multidisciplinary workflows, weaknesses at interfaces between teams and care settings are particularly likely to manifest as near misses rather than immediate catastrophic failure. From this perspective, near misses can be understood as successful but fragile rescue events. Failure-to-rescue following thoracic procedures is rarely attributable to operative technique alone; rather, it reflects breakdowns in postoperative surveillance, communication, and escalation pathways [16]. Near misses, therefore, represent moments when rescue succeeded narrowly, exposing vulnerabilities that, if unaddressed, may lead to future catastrophic outcomes.

Traditional morbidity and mortality conferences, despite their central role in surgical education, often reinforce individual-centered narratives. Case discussions may implicitly focus on identifying where a clinician might have acted differently, with less systematic attention to workflow design, communication structures, staffing models, or cognitive load. As a result, similar near misses may recur across different providers and institutions, each time framed as a new event rather than as evidence of a persistent system vulnerability [8,9]. Quality improvement seeks to address this limitation by shifting the analytic question from “who erred” to “what conditions made this outcome possible, and how might they recur?” [3,4].

The relevance of this shift is underscored by contemporary surgical quality frameworks. Of note, these observations are consistent with a broad body of international surgical quality literature demonstrating that differences in postoperative mortality are driven primarily by variation in system response to complications rather than complication incidence itself, across multiple surgical disciplines and healthcare systems [16,17,20]. Failure to rescue following thoracic procedures is rarely attributable to operative technique alone; instead, it reflects the collective capacity of the system to recognize complications early, mobilize resources, and coordinate timely responses across disciplines and care environments [16]. Similarly, composite metrics such as textbook outcome demonstrate that adverse results often arise from cumulative process failures rather than singular intraoperative events [26]. Near misses occupy a critical position within these frameworks, representing moments when system vulnerabilities were exposed but effective recovery prevented irreversible harm.

Reframing near misses as indicators of system vulnerability rather than individual failure expands the scope of professional responsibility. Surgeons remain accountable for technical excellence and clinical judgment, but they also become active participants in system design, feedback, and improvement. This perspective aligns accountability with patient safety rather than blame and enables proactive identification of risk before catastrophic outcomes occur [3,4]. In thoracic surgery, where adaptability and recovery often determine outcomes, such a systems-based approach provides the conceptual foundation for transforming near misses from uncomfortable anecdotes into powerful drivers of improvement.

## 4. Near Misses as Informative Signals for System-Level Learning

### 4.1. Recognition

**Figure 1:**
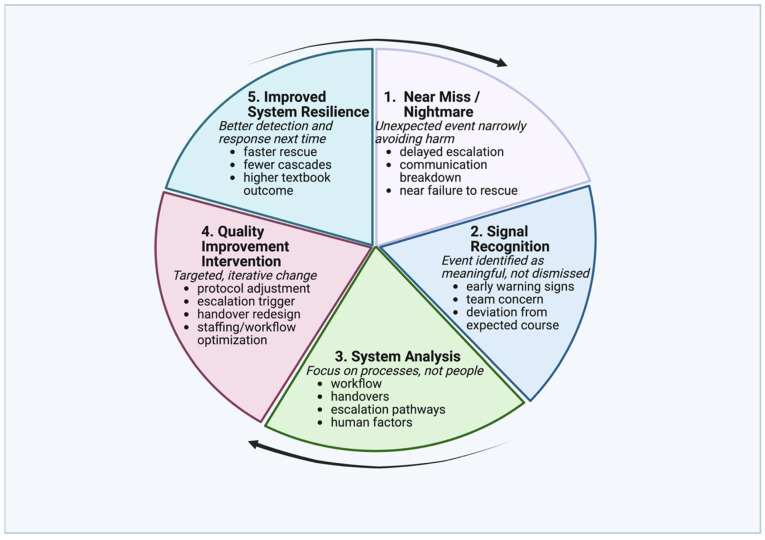
Conceptual learning loop linking near misses to system resilience in thoracic surgery. Improved system resilience reduces the likelihood and severity of harm but does not eliminate near-miss events, as variability, uncertainty, and adaptive human performance are inherent features of complex surgical systems. Created in BioRender. Magouliotis, D. (2026) https://BioRender.com/08ochqs (accessed on 4 January 2026).

Barriers to recognition include ambiguous monitoring thresholds and normalization of deviation, while facilitators include standardized early warning systems and multidisciplinary situational awareness. Near misses represent a unique category of clinical events that occur at the interface between safe recovery and adverse outcome. Figure 1 illustrates a conceptual learning loop through which near misses in thoracic surgery are recognized, analyzed, and translated into targeted quality improvement interventions that strengthen system resilience. In thoracic surgery, postoperative deterioration often presents initially with subtle respiratory or hemodynamic changes, making early recognition particularly challenging. Near misses capture these early warning failures and adaptive responses, offering insight into escalation thresholds, team communication, and system readiness before irreversible harm occurs. Unlike major complications, they do not result in durable patient harm and therefore rarely appear in traditional outcome registries or quality dashboards. Nevertheless, near misses provide critical insight into how surgical systems function under stress, capturing early breakdowns in monitoring, communication, escalation, and coordination before irreversible failure occurs [8,9]. From a quality improvement perspective, near misses should be viewed not as inconsequential “close calls,” but as informative signals that reveal latent system vulnerabilities embedded within routine care delivery.

### 4.2. Analysis

Near-miss events provide early insight into system performance before irreversible harm occurs. Conventional approaches to surgical quality measurement emphasize endpoints such as mortality, major morbidity, and readmission. While these outcomes remain essential, they provide limited visibility into upstream processes and decision points that shape patient trajectories. Near misses occur earlier along the failure cascade and therefore illuminate the dynamic interactions between clinicians, workflows, and organizational structures that determine whether deterioration is recognized and addressed in time [5,7]. When systematically aggregated, near misses expose recurring patterns—delayed escalation, ambiguous responsibility, information loss during handovers, or mismatches between workload and staffing—that are largely invisible to outcome-based surveillance alone [5,8]. Representative near-miss scenarios encountered in thoracic surgery, the underlying system vulnerabilities they expose, and corresponding quality improvement strategies are outlined in Table 2**.** Quantitative estimates of near-miss frequency are currently limited by the absence of standardized reporting frameworks and institutional variability.

In thoracic surgery, the value of these early signals is particularly pronounced. Postoperative deterioration often evolves rapidly and non-linearly, with subtle physiological changes preceding abrupt decompensation. Near misses capture moments of diagnostic uncertainty and adaptive response, offering insight into how teams interpret risk and prioritize action in real time. Importantly, these events frequently reflect successful recovery rather than failure, highlighting the mechanisms through which harm was avoided despite system stress [16,25]. Understanding why recovery occurred—rather than focusing solely on why failure occurs—provides a more complete picture of system performance.

Despite their analytic value, near misses are frequently underreported and poorly utilized. Hierarchical culture, fear of reputational harm, and limited feedback from incident-reporting systems discourage systematic documentation, reinforcing the perception that near misses are anecdotal or administratively burdensome [9]. Quality improvement reframes this perspective by treating near misses as structured data rather than narrative events. The objective is not to catalog individual errors, but to identify system conditions that repeatedly place patients at risk and to target these conditions for redesign [3,4].

### 4.3. Intervention

Effective system-level learning from near misses requires deliberate infrastructure. High-performing surgical programs integrate near-miss analysis into routine clinical workflows through standardized debriefings, focused multidisciplinary reviews, and targeted audits that emphasize process and context rather than individual performance [3,4]. Closing the feedback loop is critical: when clinicians observe that near-miss reporting leads to tangible changes in protocols, escalation pathways, or resource allocation, engagement and reporting increase, reinforcing a culture of learning rather than blame.

### 4.4. Feedback

The relevance of near misses as system-level signals is further underscored by contemporary quality frameworks such as failure to rescue and textbook outcome. These constructs explicitly acknowledge that surgical quality is determined not only by the occurrence of complications, but by the system’s capacity to recognize deterioration, coordinate timely responses, and support recovery [16,26]. Near misses occupy a central position within these paradigms, representing instances in which rescue succeeded narrowly. Systematic analysis of such events provides actionable insight into how resilience is generated in everyday practice.

By reframing near misses as informative signals rather than dramatic anecdotes, quality improvement shifts its focus from rare catastrophic failures to routine system performance. Even in highly resilient systems, near misses continue to occur because complexity and uncertainty cannot be fully eliminated; resilience determines whether these events lead to harm or recovery. This perspective aligns with modern safety science, which emphasizes learning from adaptation, recovery, and successful performance under variability [23,24]. In thoracic surgery, where uncertainty is inherent and margins for error are narrow, systematic learning from near misses offers a powerful mechanism for improving reliability, strengthening resilience, and preventing future harm.

## 5. A Practical Framework for Learning from near Misses in Thoracic Surgery

To translate conceptual insights into clinical practice, near-miss learning in thoracic surgery should follow a structured yet flexible framework encompassing reporting, review, and action.

Reporting: Near misses should be captured through simplified, non-punitive reporting mechanisms integrated into routine clinical workflows. Emphasis should be placed on events involving delayed recognition, escalation, or coordination rather than technical error alone. Anonymous or team-based reporting may enhance engagement and reduce fear of blame.

Review: Reported near misses should be reviewed in focused, multidisciplinary forums distinct from traditional morbidity and mortality conferences. Analysis should prioritize system conditions, communication pathways, workload, and handover processes rather than individual performance. Aggregating similar events over time allows identification of recurrent vulnerabilities that may not be apparent from isolated cases.

Action: Insights derived from near-miss analysis should be translated into targeted quality improvement interventions, such as clarification of escalation thresholds, redesign of monitoring protocols, or standardization of handover practices. Closing the feedback loop is essential; clinicians must observe that reporting near misses leads to tangible system change in order to sustain engagement and learning.

## 6. Culture and Leadership as Determinants of Quality Improvement Success

Among the multiple components of quality improvement in thoracic surgery, organizational culture and leadership represent the most influential—and the most challenging—determinants of success. While protocols, pathways, and metrics can be formally implemented, their effectiveness depends largely on how teams interpret risk, communicate uncertainty, and engage with learning following near misses and adverse events. Evidence from patient safety and organizational science consistently demonstrates that culture shapes whether system vulnerabilities are exposed and addressed or instead normalized and concealed [10,11,12,13].

Surgical culture has traditionally emphasized autonomy, decisiveness, and individual accountability—attributes that are essential in high-stakes operative environments but that may inadvertently suppress open discussion of uncertainty and vulnerability. Near misses that resolve without patient harm are often reframed as demonstrations of individual competence or intuition rather than as indicators of system stress. Over time, this normalization of deviance reduces vigilance and increases tolerance for unsafe conditions, thereby narrowing margins of safety [7]. For example, in thoracic surgery, repeated acceptance of borderline postoperative hypoxia without escalation following lung resection may gradually become normalized, until a similar pattern culminates in delayed rescue and major respiratory morbidity. In thoracic surgery, where patient trajectories can deteriorate rapidly, such cultural dynamics may delay escalation and impede collective response.

Psychological safety has emerged as a critical enabling condition for effective learning from near misses. Teams characterized by psychological safety are more likely to report concerns, question assumptions, and engage in reflective discussion without fear of blame or humiliation [10,11,12,13]. In these environments, near misses are interpreted as shared learning opportunities rather than personal failures. Conversely, in cultures dominated by hierarchy or punitive responses, near misses remain underreported, limiting the system’s ability to detect recurring vulnerabilities.

Leadership behavior plays a decisive role in shaping this environment. Cultural change is driven less by formal policy than by everyday actions modeled by senior clinicians. When attending surgeons openly acknowledge uncertainty, discuss their own near misses, and actively solicit input from multidisciplinary team members, they signal that learning and patient safety take precedence over individual image. Conversely, dismissive responses to concerns—particularly subtle or indirect ones—can rapidly undermine trust and suppress reporting [11,13]. Effective leaders, therefore, function not only as technical experts but also as stewards of psychological safety and system learning.

Aligning incentives with learning is equally important. When quality assessment focuses narrowly on outcomes such as mortality or complication rates, teams may become risk-averse or selective in reporting events that could be perceived negatively. In contrast, frameworks such as failure to rescue and textbook outcome implicitly reward early recognition, coordinated response, and recovery from complications, reinforcing behaviors that support transparency and resilience [16,26]. These metrics shift the narrative of quality from avoidance of failure to mastery of response, creating space for meaningful engagement with near misses.

Resistance to cultural change is inevitable and should be anticipated. Surgeons may perceive quality improvement initiatives as threats to autonomy or as administrative burdens disconnected from clinical reality. Addressing these concerns requires reframing quality improvement as an extension of professional responsibility and surgical craftsmanship rather than external oversight. Just as technical skill improves through feedback and repetition, system performance improves through reflection, adaptation, and shared learning [3,4].

Ultimately, culture determines whether near misses are buried or transformed into drivers of improvement. In environments that prioritize learning, near misses become reference points that enhance collective vigilance and preparedness. In environments characterized by fear or rigid hierarchy, the same events are forgotten, only to re-emerge later with greater consequence. For thoracic surgery, where resilience often determines outcomes, culture and leadership are not ancillary to quality improvement—they are foundational.

## 7. Translating Quality Improvement into Prevention of Future Harm

The ultimate objective of quality improvement in thoracic surgery is not conceptual refinement, but the prevention of recurrent harm. Near misses only achieve their full value when they lead to changes that meaningfully alter future clinical trajectories. In this sense, quality improvement functions as a translational discipline, converting experiential insight into protective system design [3,4]. Programs that succeed in this translation do so not through large-scale reform, but through targeted, iterative interventions informed by recurring patterns identified through near-miss analysis.

In thoracic surgery, many of the most effective preventive interventions are modest in scope yet substantial in impact. Importantly, the impact of near-miss–driven interventions can be evaluated using objective performance metrics, including failure-to-rescue rates, intensive care unit length of stay, unplanned readmissions, and composite quality indicators such as textbook outcome. Linking near-miss learning to these measurable endpoints allows quality improvement efforts to be systematically monitored, benchmarked across institutions, and refined over time. Clarification of escalation thresholds, standardization of postoperative monitoring, redesign of handover processes, and explicit delineation of responsibility during periods of clinical deterioration have all been shown to reduce delays in recognition and response [5,8]. When these interventions are informed by near misses rather than catastrophic outcomes, they are more precisely targeted to points of real-world vulnerability and more likely to be adopted by frontline clinicians.

The relevance of this approach is particularly evident in the context of failure to rescue. Complications following thoracic procedures are often unavoidable; what distinguishes outcomes across institutions is the system’s capacity to detect deterioration early and coordinate timely, effective responses [16]. Near misses frequently represent successful rescues that occurred at the limits of system performance. Systematic analysis of these events allows teams to identify which signals prompted action, which communication pathways functioned effectively, and where margins of safety were narrow. Such insight enables proactive strengthening of rescue capacity before failure occurs.

Composite quality metrics such as textbook outcomes further illustrate how quality improvement can shift focus from isolated events to integrated care pathways. Textbook outcome captures the cumulative performance of perioperative care, including avoidance of major complications, timely recovery, and coordinated multidisciplinary management [26]. Near misses illuminate the spaces between these components—moments when processes nearly failed and where targeted intervention can improve reliability across the entire pathway. By linking near-miss analysis to composite metrics, quality improvement aligns local learning with measurable system performance.

Sustained prevention of future harm also depends on organizational memory. Without mechanisms for documentation, synthesis, and dissemination, lessons derived from near misses remain confined to individual experience and are lost with staff turnover or changing clinical contexts. Quality improvement externalizes this memory by embedding insights into protocols, checklists, escalation algorithms, and shared mental models that persist beyond individual cases [22]. In doing so, organizations transition from reactive learning to anticipatory system design.

Importantly, effective prevention does not require rigid standardization. Thoracic surgery demands adaptability, and excessive protocolization may introduce new risks. High-performing systems balance consistency with flexibility, standardizing responses to predictable threats while preserving clinical judgment in complex or novel situations [23,24]. Near-miss analysis helps delineate this balance, identifying where variability is beneficial and where it is hazardous.

Over time, the cumulative effect of these efforts is a shift in how risk is experienced and managed. Near misses become less frequent not because uncertainty has been eliminated, but because systems are better equipped to detect, absorb, and respond to it. In thoracic surgery, where the margin between recovery and deterioration can be narrow, this capacity for early detection and recovery represents a central determinant of patient safety. Quality improvement, grounded in systematic learning from near misses, thus serves as a primary mechanism for preventing future harm and improving the reliability of surgical care. The integration of near-miss–informed learning with contemporary surgical performance metrics and quality frameworks is summarized in the preceding sections.

## 8. Conclusions

Near misses represent a critical yet underutilized source of insight in thoracic surgery. Far from being inconsequential “close calls,” these events occur at the boundary between recovery and harm and expose latent vulnerabilities in monitoring, communication, escalation, and coordination that shape patient outcomes [5,6,7]. While several illustrative examples are drawn from thoracic surgery—specific analyses by the authors and collaborators, these findings align closely with broader international evidence from surgical quality research and patient safety science. This narrative review highlights that near misses provide uniquely informative signals of everyday system performance, offering opportunities for intervention well before catastrophic failure occurs. The evidence synthesized here supports a shift in how surgical quality is conceptualized and assessed. Traditional reliance on mortality and complication rates alone fails to capture the dynamic processes that determine whether deterioration is recognized and addressed in time. Contemporary frameworks such as failure to rescue and textbook outcome more accurately reflect the realities of thoracic surgical care by emphasizing recovery, coordination, and system responsiveness rather than complication avoidance alone [16,26]. Near misses occupy a central position within these paradigms, representing moments when effective rescue was possible but not assured. Importantly, the ability to learn from near misses is not primarily a technical challenge, but a cultural one. Psychological safety, leadership behaviors, and organizational commitment to learning determine whether near misses are reported, analyzed, and translated into durable improvement [10,11,12,13,27]. Without these conditions, even well-designed quality improvement initiatives are unlikely to achieve sustained impact. Future efforts should focus on integrating near-miss analysis into routine quality improvement infrastructure, linking local learning to system-level metrics, and developing standardized yet flexible approaches to escalation and response. Specifically, three complementary implementation strategies may facilitate translation into practice. First, near-miss reporting and review should be formally embedded into departmental quality improvement meetings and safety huddles, distinct from traditional morbidity and mortality conferences. Second, near-miss data should be linked to established performance metrics such as failure-to-rescue rates and textbook outcome dashboards to enable monitoring of system resilience over time. Third, multicenter thoracic surgery collaborations and registries could incorporate structured near-miss surveillance to enable benchmarking, shared learning, and development of best practices across institutions. Further research is also needed to better characterize near-miss patterns specific to thoracic surgical populations and to evaluate the impact of near-miss–driven interventions on patient-centered outcomes. Ultimately, excellence in thoracic surgery should be defined not by the absence of complications, but by the capacity of surgical systems to learn, adapt, and prevent future harm through systematic engagement with what almost went wrong.

## Figures and Tables

**Table 1:** Conceptual Framework for Understanding Near Misses in Thoracic Surgery.

Domain	Traditional Interpretation	Systems-Based Interpretation	Implications for Quality Improvement
Definition of near miss	Fortunate avoidance of harm	Early signal of system vulnerability	Opportunity for proactive intervention
Primary contributing mechanism	Human performance variability within system constraints	Interaction of people, processes, and context	Shift focus from blame to system redesign
Learning trigger	Catastrophic complication	Everyday recovery under stress	Learning before irreversible harm
Unit of analysis	Single case or decision	Patterns across workflows and teams	Identification of recurrent vulnerabilities
Role in quality assessment	Marginal or ignored	Central indicator of system performance	Integration into QI surveillance

**Table 2:** Common Near-Miss Scenarios in Thoracic Surgery and Targeted Quality Improvement Interventions. Approximate frequencies are not reported because near-miss events are not systematically captured across institutions and available data are highly heterogeneous.

Near-Miss Scenario	Clinical Context	Underlying System Vulnerability	Targeted Quality Improvement Strategy
Delayed recognition of respiratory deterioration	Post-lung resection hypoxia	Ambiguous monitoring thresholds; unclear escalation	Standardized early warning criteria; escalation triggers
Near failure to rescue	Late response to postoperative complication	Fragmented communication across care settings	Multidisciplinary rescue protocols
Communication breakdown during handover	OR–ICU or ICU–ward transfer	Information loss; lack of shared mental model	Structured handover tools
Workflow overload	High census or staffing variability	Cognitive overload; competing priorities	Workflow redesign; protected high-risk review
Normalization of deviance	Recurrent close calls	Cultural acceptance of unsafe workarounds	Leadership-led near-miss review

Abbreviations: OR = Operating Room; ICU = Intensive Care Unit.

## Data Availability

No new data were created or analyzed in this study.
